# Photoelectrolysis Using Type-II Semiconductor Heterojunctions

**DOI:** 10.1038/s41598-017-11971-x

**Published:** 2017-09-14

**Authors:** S. Harrison, M. Hayne

**Affiliations:** Department of Physics, Lancaster University, LA1 4YB Lancaster, UK

## Abstract

The solar-powered production of hydrogen for use as a renewable fuel is highly desirable for the world’s future energy infrastructure. However, difficulties in achieving reasonable efficiencies, and thus cost-effectiveness, have hampered significant research progress. Here we propose the use of semiconductor nanostructures to create a type-II heterojunction at the semiconductor–water interface in a photoelectrochemical cell (PEC) and theoretically investigate it as a method of increasing the maximum photovoltage such a cell can generate under illumination, with the aim of increasing the overall cell efficiency. A model for the semiconductor electrode in a PEC is created, which solves the Schrödinger, Poisson and drift–diffusion equations self-consistently. From this, it is determined that ZnO quantum dots on bulk n-InGaN with low In content *x* is the most desirable system, having electron-accepting and -donating states straddling the oxygen- and hydrogen-production potentials for *x* < 0.26, though large variance in literature values for certain material parameters means large uncertainties in the model output. Accordingly, results presented here should form the basis for further experimental work, which will in turn provide input to refine and develop the model.

## Introduction

An ever-increasing world population, coupled with increasing industrialisation, has led to vast increases in global demand for energy^1^. Currently, fossil fuels are used to meet most of these demands, globally accounting for 86% of primary energy consumption in 2015^2^, but our over-reliance on these fuels is fundamentally flawed: Their combustion releases atmospheric carbon dioxide, causing global climate change, and there is only a finite amount of them available for extraction^1^. We clearly need to transition to a renewable and low-greenhouse-gas energy infrastructure, and renewable hydrogen is expected to play an important role^3–5^.

Photovoltaic solar cells are currently used to convert sunlight directly into electricity, while solar thermal collectors and concentrated solar power systems produce heat as an output. The significant advantage that solar hydrogen offers over these is relative ease of storage. Hydrogen can be stored and then converted to electricity in a fuel cell as and when needed, making it particularly advantageous as a fuel for transport, or in remote communities without a mains electricity supply. Furthermore, it can be simply burned as a fuel for heating or cooking, making it a promising renewable alternative to natural gas. Indeed, plans were recently announced to make the UK city of Leeds a “hydrogen city” by 2025–30, converting the entire municipal gas network from methane to hydrogen^6^. (Though it is likely that the hydrogen will be produced by reforming natural gas, rather than by renewable, solar-powered methods.)

Renewable, carbon-neutral hydrogen can be produced by photoelectrolysis; the solar-powered splitting of water in a photoelectrochemical cell (PEC). However, despite significant research effort over the past four decades, fundamental problems still impede the progress of photoelectrolysis research toward commercial applications^4^. Namely, high efficiencies are difficult to achieve and the highest solar-to-hydrogen efficiencies to date (~18%, calculated by *ν* = (1.23 V) (J_opp_)/*P*_in_, where *J*_opp_ is the rate of hydrogen production converted to a current density, and *P*_in_ is the incident solar irradiance^4^) utilise complex and costly PEC designs (e.g., multi-junction cells) or are based on rare-Earth materials^7^, while cost-effective devices (e.g., utilising nanoparticles) rarely demonstrate efficient water-splitting capability^8^. For research to progress, innovation in both materials development and device design is clearly needed. In this paper, we present theoretical calculations into the novel use of type-II semiconductor nanostructures at the semiconductor–electrolyte interface (SEI), with a view to increasing the maximum photovoltage that can be generated in a PEC.

## Theory

It is important to note that the development of solid-state physics and electrochemistry as separate research fields has led to the overlapping definition of the terms Fermi level, in reference to the electrode, and redox potential, in reference to the electrolyte. The difference between these two terms is purely conceptual; they both refer to the chemical potential of the electron^9^. To avoid confusion, the convention of referring to all chemical potentials as Fermi levels will be employed herein, with the Fermi level in the electrolyte referred to as the redox Fermi-level *E*_F,redox_ and the Fermi level in the semiconductor referred to as the semiconductor Fermi-level *E*_F,s_.

Another conflicting convention is that electrochemists tend to indicate the position of energy levels and potentials as a potential relative to the standard hydrogen electrode (SHE), while solid-state physicists do so as an energy relative to, for example, the vacuum level. This means that an energy level that is *higher* in potential is *lower* in energy (*E* = −*eV*, where *E* is energy, *e* is electron charge and *V* is potential). Throughout this work, unless otherwise indicated, we state energy levels and potentials relative to the vacuum.

Photogenerated electrons and holes in a semiconductor electrode are split up by a built-in electric field, which is generated by band bending at the SEI. These carriers are driven to either the SEI or the counter-electrode–electrolyte interface to transfer to the electrolyte and drive hydrogen- and oxygen-evolution reactions.

**Figure 1:**
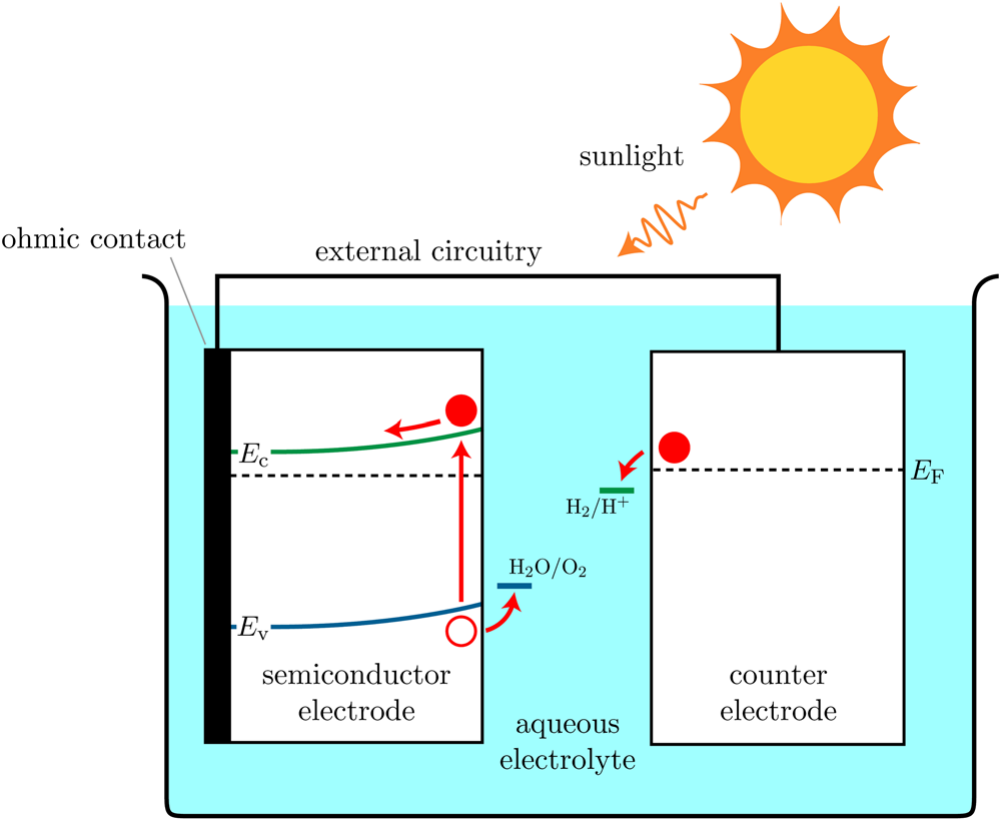
A representation of a photoelectrochemical cell (PEC) with an n-type semiconductor electrode. Shown in the electrodes are the conduction (green line) and valence (blue line) band edges, *E*_c_ and *E*_v_ respectively, and the equilibrium Fermi-level *E*_F_ (dashed line). The oxygen- and hydrogen-production potentials *E*_ox_ (H_2_O/O_2_) and *E*_red_ (H_2_/H^+^) respectively are shown in the electrolyte.

Band bending occurs due to discontinuity between the semiconductor Fermi-level and the redox Fermi-level: In the case of an n-type semiconductor, the semiconductor Fermi-level is usually greater than the redox Fermi-level (*E*_F,s_ > *E*_F,redox_) and electrons flow from the semiconductor to the electrolyte in an attempt to equilibrate the interface, such that the semiconductor Fermi-level moves down in energy until it equates with the redox Fermi-level; *E*_F,s_ = *E*_F,redox_. This results in upward band bending and a depletion layer near the surface of the semiconductor, as illustrated in Fig. 1. Conversely, p-type semiconductors usually have *E*_F,redox_ > *E*_F,s_, resulting in downward band bending at the SEI. Though p-type semiconductors are generally more stable in a PEC (due to a Fermi level at a higher energy, and thus a redox potential at a lower potential), only n-type semiconductors are considered herein as the electron-confining type-II heterojunction required for water splitting to occur in a p-type PEC is much more difficult to achieve than the hole-confining heterojunction required in an n-type PEC.

**Figure 2:**
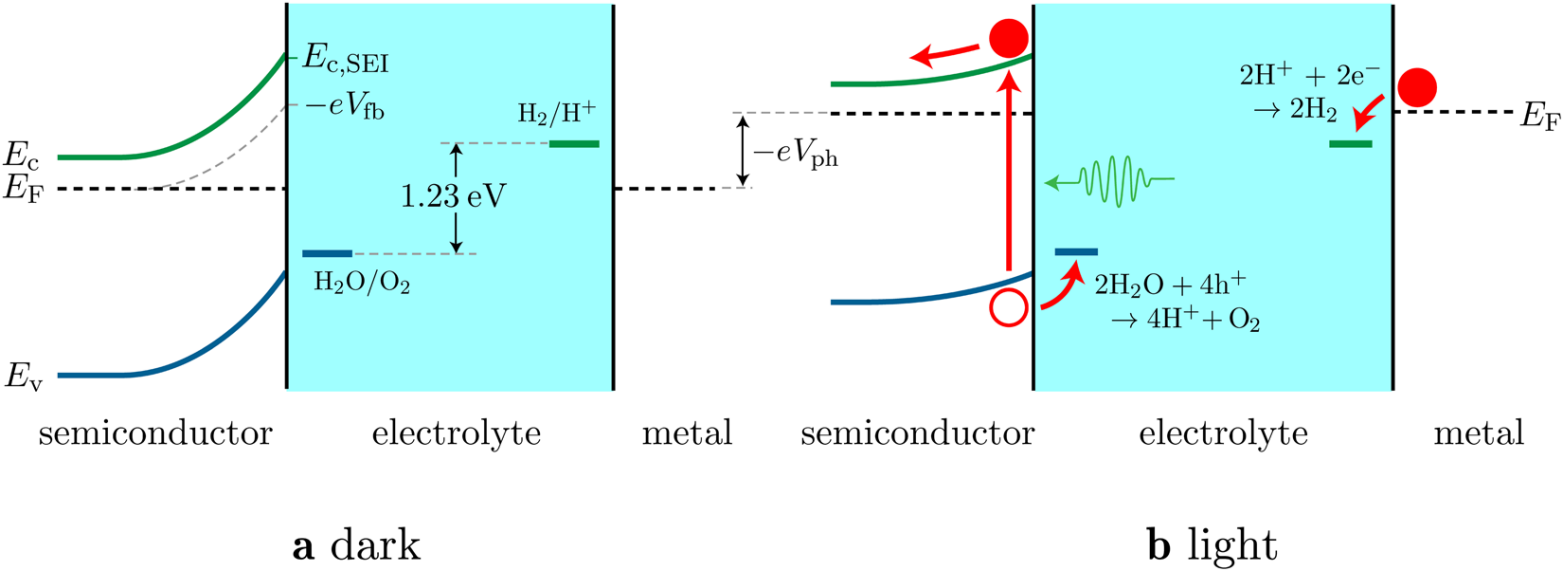
Energy band diagram for a PEC with an n-type semiconductor electrode **(a)** in the dark and **(b)** illuminated. Incident sunlight generates a photovoltage *V*_ph_ that raises the semiconductor and hence counter-electrode Fermi-level, so that it and the valence band edge at the semiconductor–electrolyte interface (SEI) straddle the oxygen- and hydrogen-production potentials, which are shown in the electrolyte. Holes and electrons can then transfer to the solution to drive water-splitting half-reactions, shown in **(b)**. The conduction band edge at the SEI *E*_c,SEI_ and the flat-band potential *V*_fb_ are also shown.

Under illumination, this flow of photogenerated carriers causes a photovoltage *V*_ph_, which has the effect of raising the energy of the semiconductor and counter-electrode Fermi-levels (which are equal), thereby reducing the band bending. If they are raised sufficiently so that they are above the energy of the hydrogen-production potential *E*_red_, then electrons transfer from the counter electrode to the electrolyte and drive hydrogen production. Similarly, if the valence band edge at the SEI is lower than the energy of the oxygen-production potential *E*_ox_, then holes transfer to the electrolyte to drive oxygen production, as shown in Fig. 2. The hydrogen- and oxygen-production potentials are given by ref. 10:

1 $$\begin{array}{cc}{E}_{{\rm{red}}}=-0.059\times {\rm{pH}} & {\rm{vs}}{\rm{.}}\,{\rm{SHE}}\,{\rm{at}}\,{\rm{25}}\,^\circ {\rm{C}}\end{array}$$

and

2 $$\begin{array}{cc}{E}_{{\rm{ox}}}=1.229-0.059\times {\rm{pH}} & {\rm{vs}}{\rm{.}}\,{\rm{SHE}}\,{\rm{at}}\,{\rm{25}}\,^\circ {\rm{C}}.\end{array}$$

Given sufficient illumination, the bands will fully flatten and the potential of the semiconductor Fermi-level at which this occurs is called the *flat-band potential V*_fb_, such that

3 $$\begin{array}{c}-e{V}_{{\rm{fb}}}={E}_{{\rm{c}},{\rm{SEI}}}-|{E}_{{\rm{c}}}-{E}_{{\rm{F}}}|,\end{array}$$

where *E*_c,SEI_ is the conduction band edge at the SEI, *E*_*c*_ is the bulk conduction band edge, and *E*_*F*_ is the equilibrated Fermi-level. Without band bending, no photocurrent and thus no photovoltage, can be generated to further raise the Fermi levels: the flat-band potential is the highest possible energy that the semiconductor Fermi-level, and therefore the counter-electrode Fermi-level, can reach under illumination. Hence this dictates whether or not a particular n-type semiconductor has the ability to reduce water to hydrogen.

We can summarise the above into the condition that, for water splitting to occur in a PEC with an n-type semiconductor electrode, the valence band edge at the SEI must be lower in energy than the oxygen-production potential, and the flat-band potential −*eV*_fb_ must be higher in energy than the hydrogen-production potential. In addition, overpotentials for both the hydrogen and oxygen half-reactions are needed, of around 0.275 and 0.050 V respectively^11, 12^. Furthermore, photo-excitation of electrons is to the conduction band and there is therefore an energy loss of *E*_*c*_ − *E*_*F*_ when they move down to the Fermi level in the counter electrode, which is typically 0.05–0.2 V, depending on the material and doping^10^. With these factors taken into account, the 1.229 eV gap between the oxygen- and hydrogen-production potentials (shown in Fig. 2) results in the need for a band gap of approximately 1.8 eV. The optimal band gap is, of course, a trade-off between maximising solar absorption while meeting the aforementioned criteria; below 400 nm, there is a large drop in the intensity of solar radiation, and thus our semiconductor band gap should be ≥ 1.8 eV and considerably less than ~3.1 eV.

## Type-II nanostructures at the semiconductor–electrolyte interface

Finding materials with sufficiently positioned flat-band potentials is a major bottleneck to photoelectrolysis research, and PECs meeting this criterion either have large band gaps and are therefore inefficient at absorbing sunlight^13, 14^, or are based on complex and costly multi-junction designs^4, 15^. Here, we propose the novel use of type-II semiconductor nanostructures at the SEI to limit the flattening of bands under illumination and thus increase the maximum photovoltage that can be generated.

**Figure 3:**
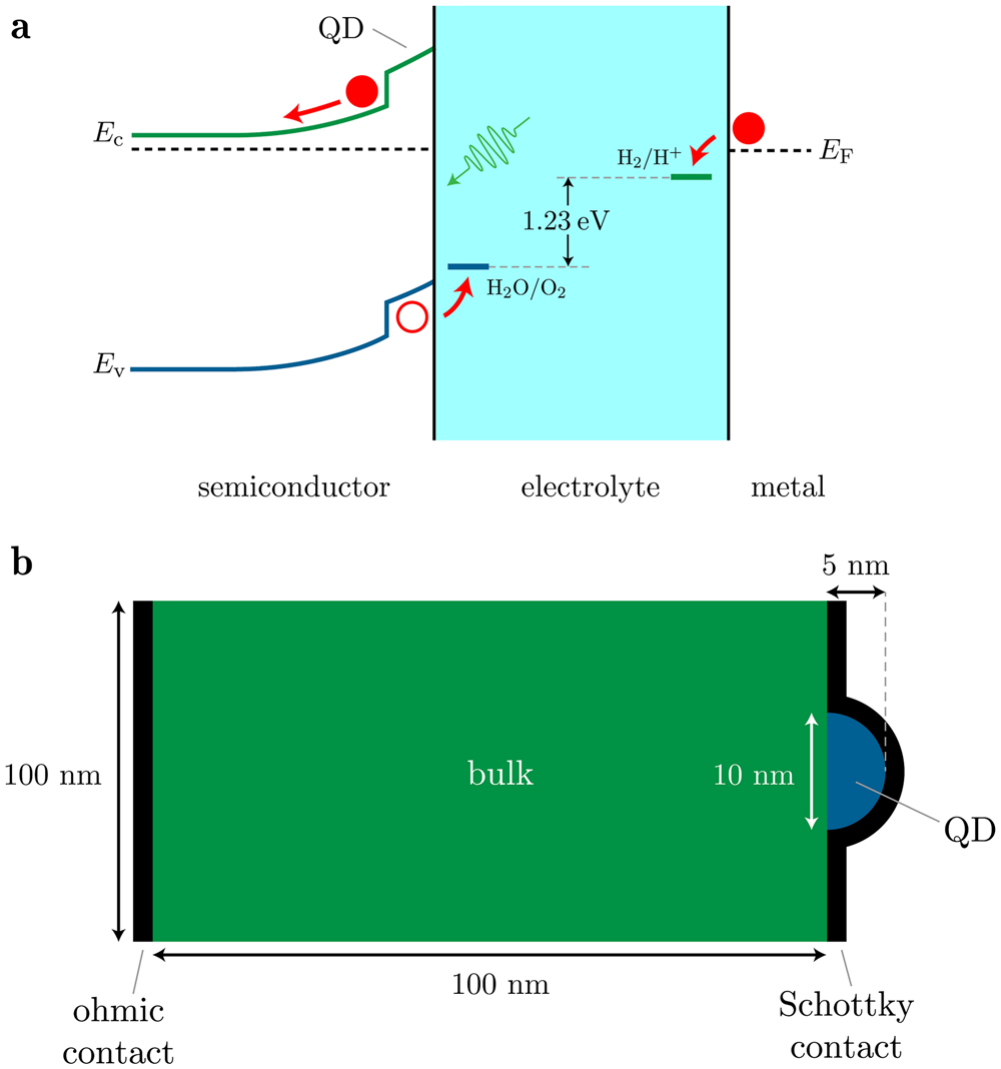
**(a)** Ideal band structure of a PEC utilising type-II quantum dots (QDs) at the SEI to limit the flattening of the bands due to the photogenerated current. The band structure represents the energy levels in 1D slice through the center of a QD. **(b)** A representative 2D semiconductor electrode model with a QD included at the SEI.

Type-II systems have band alignments such that one carrier is confined, while the other is free to roam in the bulk material. Consider the placement of hole-confining quantum dots (QDs) at the SEI: Upon illumination, excitons are generated near the surface of the semiconductor and soon split up by the built-in electric field. For an n-type semiconductor, electrons flow to the counter electrode, while holes travel toward the QDs at the SEI. If the QDs offer a suitable confining potential, holes may become trapped. This excess of positive charges at the SEI will raise energy levels at the interface (but not in the bulk semiconductor), thus increasing the band bending and countering the effect that the flow of carriers has in flattening the bands. This will result in a larger Schottky barrier (*ϕ*_B_ = *E*_c,SEI_ − *E*_*F*_) and therefore increase the maximum photovoltage that the PEC can generate, maximising the likelihood that the semiconductor (and therefore, counter-electrode) Fermi-level will be pushed above the energy of the hydrogen-production potential. An ideal schematic of the band structure of such a system under illumination is shown in Fig. 3a. Herein, we present theoretical calculations of the confined-carrier energy levels for a number of type-II heterostructures, demonstrating the practical feasibility of such a system. Figure 3b is a representative example of the 2D semiconductor electrodes modelled, for which the Schrödinger, Poisson and drift–diffusion equations are solved self-consistently, taking into account strain. Full details of the modelling methodology can be found in the Methods section.

## Results

Recently, In_*x*_Ga_1−*x*_N has shown great promise as a bulk material to split water, with suitably positioned band edges, in part due to a tuneable band gap, high carrier mobility and good chemical stability^10, 16, 17^. Furthermore, the band alignment of n-InGaN relative to potential QD materials means fabricating a hole-confining type-II system is easily achievable, and thus n-InGaN will be used as a substrate material.

**Figure 4:**
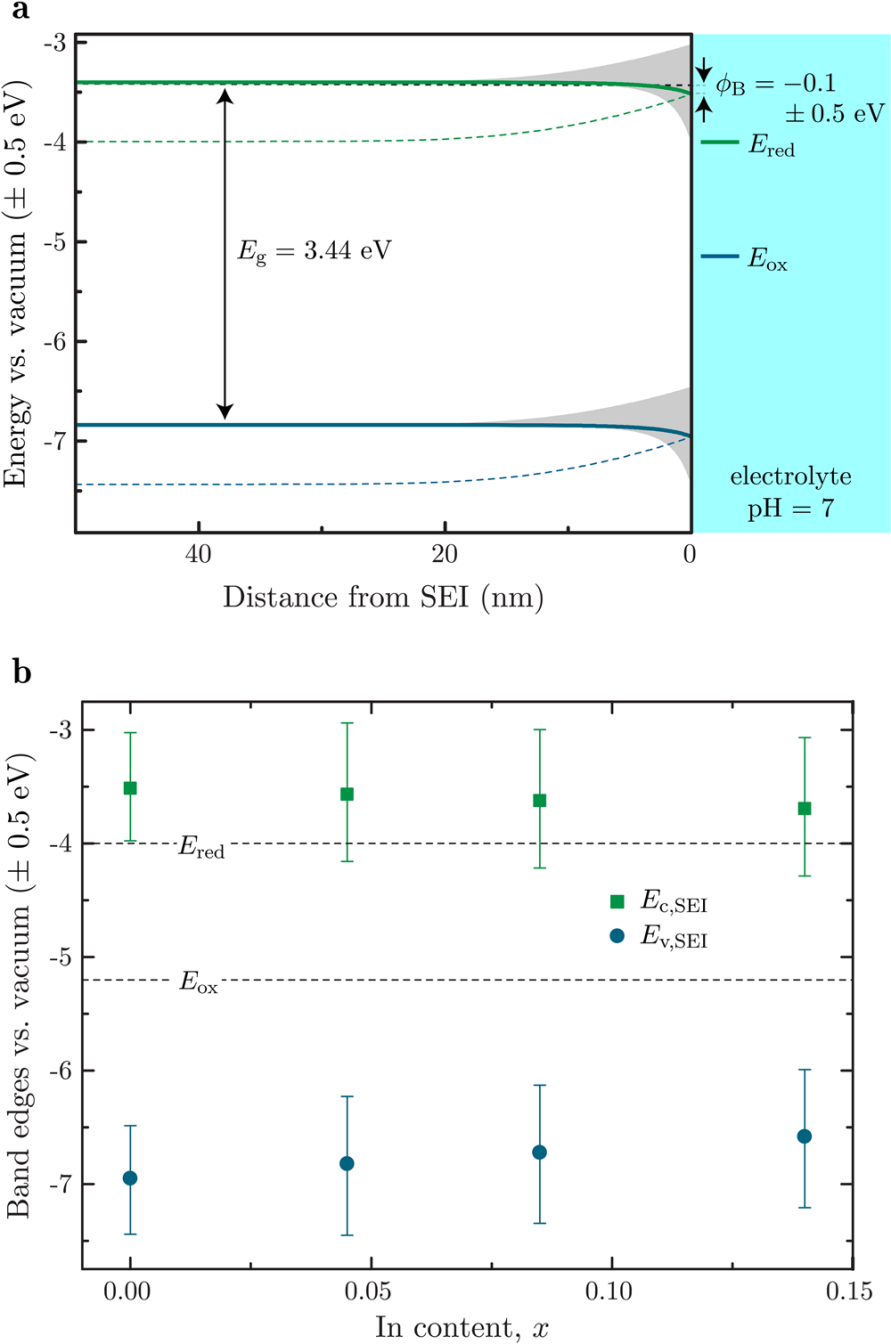
**(a)** Simulated band structure of a wurtzite n-GaN semiconductor electrode in water (pH = 7) with a dopant (Si) concentration of 10^18^ cm^−2^. For *ϕ*_B_ = −0.1 ± 0.5 eV (deduced from *χ* = 3.4 ± 0.5 eV and *V*_fb_ = 3.5 ± 0.2 V vs. vacuum), the conduction band edge is shown as a green solid line, the (heavy hole) valence band edge as a blue solid line and the Fermi energy as a dashed black line. The uncertainties in the band edge values are shaded in grey. The value of *ϕ*_B_ = 0.77 eV from literature gives us *χ* = 4.0 ± 0.2 eV, and the subsequent conduction and valence band edges are shown as dotted lines (uncertainties omitted for clarity). Band edges are positioned relative to the vacuum by *χ*, and it is this that gives rise to the ±0.5 eV uncertainty in the energy axis. The hydrogen- and oxygen-production potentials are positioned using equations (1) and (2), such that *E*_red_ = 4.0 ± 0.2 eV vs. vacuum and *E*_ox_ = −5.2 ± 0.2 V vs. vacuum. The error arises from their conversion to values relative to the vacuum from values relative to the standard hydrogen electrode (SHE). **(b)** Conduction and (heavy) hole valence band edges at the SEI, *E*_c,SEI_ and *E*_v,SEI_, for varying In content *x*. Values for *V*_fb_ were taken from ref. 10 and *ϕ*_B_ was calculated using these and a non-linear interpolation of *χ* from 3.4 ± 0.5 eV for GaN, to 5.5 ± 0.3 eV for InN, with a bowing parameter of 1.4 eV^28^.

Firstly, let us consider bulk n-GaN. Values for the electron affinity *χ* and flat-band potential *V*_fb_, both needed as input parameters for the model, vary greatly in literature; e.g., experimentally- and theoretically-determined values for *χ* range from 2.6 to 4.1 eV^18, 19^, flat-band potentials at a pH of 9 from −0.65 to −0.38 V vs. SHE, and the dependence of *V*_fb_ on pH from 45 to 71 meV per pH unit. The leads to a large variance in the height of the Schottky barrier height *ϕ*_B_, as shown in the simulated band structure presented in Fig. 4a for a pH of 7, which also includes an error of ±0.2 V for the electrode–vacuum potential conversions (again due to disagreement in literature values^20–23^). For smaller electron affinities, *ϕ*_B_ becomes negative, which seems unlikely given that water splitting has been experimentally demonstrated using GaN^24, 25^. Indeed, a value of *ϕ*_B_ ≈ 0.8 eV has been reported for various alkaline solutions^26^. Working back from the literature value of *ϕ*_B_ = 0.77 eV in KCl (pH ≈ 11)^26^, we obtain *χ* = 4.0 ± 0.2 eV. The dotted lines in Fig. 4a show the computed band structure at a pH of 7, using this value for the electron affinity, resulting in *ϕ*_B_ = 0.5 ± 0.3 eV.

Of course, it is difficult to state any conclusion with certainty from these plots, and instead they serve to highlight the complexities in accurately modelling band bending in photoelectrolytic systems. We speculate that, as water splitting has been demonstrated using GaN as an electrode^24, 25^, *χ* lies toward the higher of the literature values, but not large enough to push the conduction band edge lower than the hydrogen-production potential; a value of a little less than 4 eV seems reasonable. Here we shall use the average value from the literature of 3.4 ± 0.5 eV.

Turning our attention to In_*x*_Ga_1−*x*_N, Fig. 4b shows the calculated SEI conduction and valence band edges for differing In content *x*. A value of *χ* = 5.5 ± 0.3 eV was used for InN^19, 27^, and interpolated (with *χ* = 3.4 ± 0.5 for GaN) to give *χ* for varying *x* using a band gap bowing parameter of 1.4 eV^28^ (in the knowledge that band gap bowing is purely due to bowing of the conduction band edge^19^). *V*_fb_ for varying *x* was interpreted from ref. 10 as ~0.14 V per 10% In content increase, at a pH of 7. As expected, the valence band edge at the SEI is consistently lower in energy than the oxygen-production potential. The conduction band edge at the SEI is always higher than the energy of the hydrogen-production potential, though not within the uncertainty limits. These results are consistent with the experimental work of Beach^10^, who found only samples with *x* < 0.085 had *E*_c,SEI_ > *E*_red_ at a pH of 7. As before, uncertainties are too large to come to any definite conclusion, though it is clear that hydrogen evolution is only likely for In_*x*_Ga_1−*x*_N with low In content. Therefore, here we will consider only InGaN with *x* < 0.3, sufficiently covering the range of *x* for which water splitting is possible.

**Figure 5:**
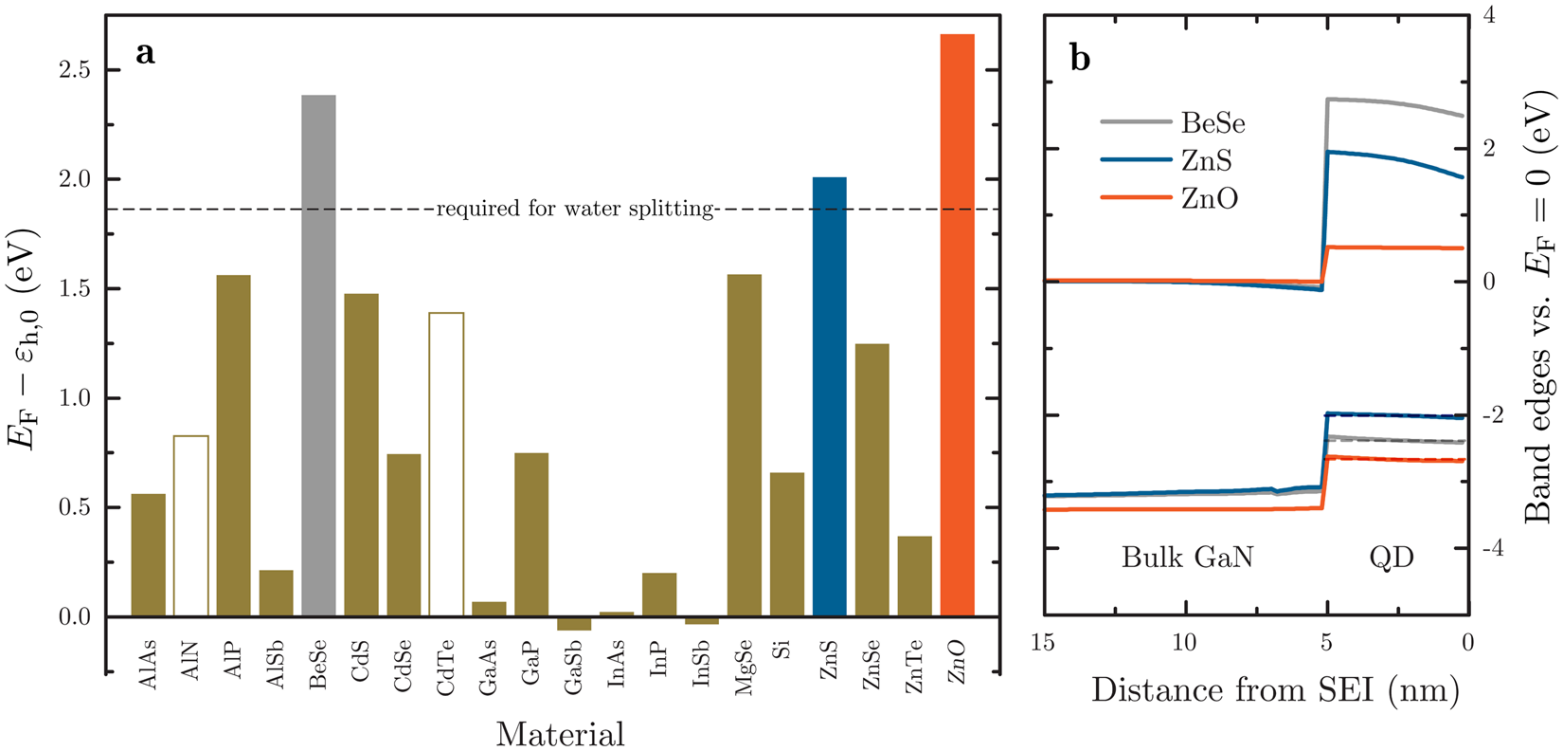
Eight-band **k** · **p** calculations with strain, for a QD fabricated from different materials, grown on n-GaN. Italics indicate that the ZnO/n-GaN system is wurtzite, while the others are zinc-blende. The data are from a 1D slice through the centre of the dot, along the growth direction. **(a)** *E*_F_ − *ε*_h,0_ for each of the different materials, of which three clearly meet the water-splitting requirement that *E*_F_ − *ε*_h,0_ > 1.8 eV. The two materials shown with hollow bars result in a type-I system, while the remaining materials are type-II (with broken-gap alignment highlighted by a negative *E*_F_ − *ε*_h,0_). **(b)** Band alignment of the three systems that meet the water-splitting requirement, showing the conduction band edge *E*_c_ and the (heavy hole) valence band edge *E*_v_ (solid lines), along with the first of the confined hole states *ε*_h,0_ (dashed lines).

Figure 5a shows *E*_F_ − *ε*_h,0_ for twenty systems with different QD materials grown on n-GaN, computed by eight-band **k** · **p** simulations with strain taken into account, where *ε*_h,0_ is the lowest heavy hole state in the QD. We can immediately exclude certain systems whose electron-donating and electron-accepting states do not straddle the hydrogen- and oxygen-production potentials by imposing the criterion that *E*_F_ − *ε*_h,0_ > 1.8 eV, slightly modifying the bulk semiconductor electrode condition that the band gap be greater than 1.8 eV. The band structures of the three materials that meet this criterion – BeSe, ZnS and ZnO – are shown in Fig. 5b.

**Figure 6:**
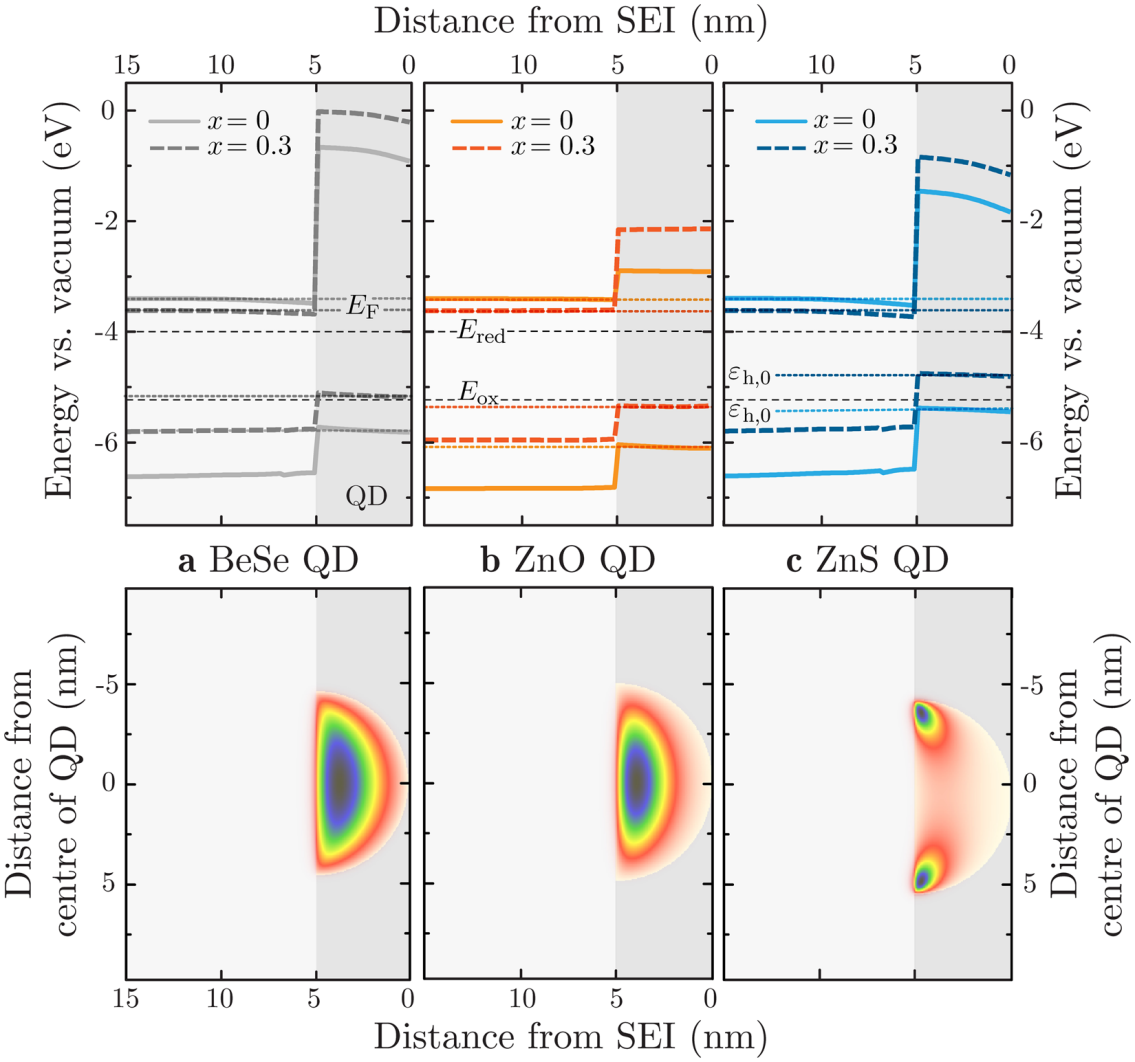
Top: Conduction and (heavy hole) valence band alignment for **(a)** BeSe, **(b)** ZnO and **(c)** ZnS QDs on In_*x*_Ga_1−*x*_N, for *x* = 0 (thick solid lines, lighter colour) and *x* = 0.3 (thick dashed lines, darker colour). The band edge data follow a line through the centre of the dot. *E*_F_ and *ε*_h,0_ are also shown (thin dotted lines), with *E*_F_ lying close to the bulk condition band edge, and *ε*_h,0_ being completely confined in the QD. The thin (black) dashed lines represent the hydrogen- and oxygen-production potentials, *E*_red_ and *E*_ox_. Uncertainties are omitted for clarity. Bottom: Contour plot of the corresponding wave function probability amplitudes for the heavy hole ground state |*ψ*_h_|^2^, overlaid on the model structure (*x* = 0). As the model is 2D, we cannot be sure whether the positioning of the probability amplitude peaks at edges of the dot, for ZnS, indicates a ring-shaped or lobed heavy-hole ground state. The former could arise because of strain energies being lower around the edge of the dot, while the latter might result from piezoelectricity.

Figure 6 shows the conduction and valence band edges for *x* = 0 and *x* = 0.3, relative to the vacuum, along with the corresponding wave function probability amplitudes for the heavy hole ground state. Uncertainties are omitted for clarity. The wave function probability amplitudes verify that the hole is strongly localised within the QD. Increasing the In content shrinks the band gap of the bulk material, making *E*_F_ − *ε*_h,0_ smaller. The optimum system is the one with the smallest possible band gap (to maximise solar absorption), while ensuring *E*_F_ and *ε*_h,0_ still straddle the hydrogen- and oxygen-production potentials, which are highlighted in the diagram. It must be remembered that here, we are simulating a system that is isolated, un-immersed in an electrolyte and without band bending at the SEI, and therefore, positions of *E*_F_ and *ε*_h,0_ are likely to be lower than when in a PEC. Critically, this could push *ε*_h,0_ above *E*_ox_, depending on the extent of the band bending.

**Figure 7:**
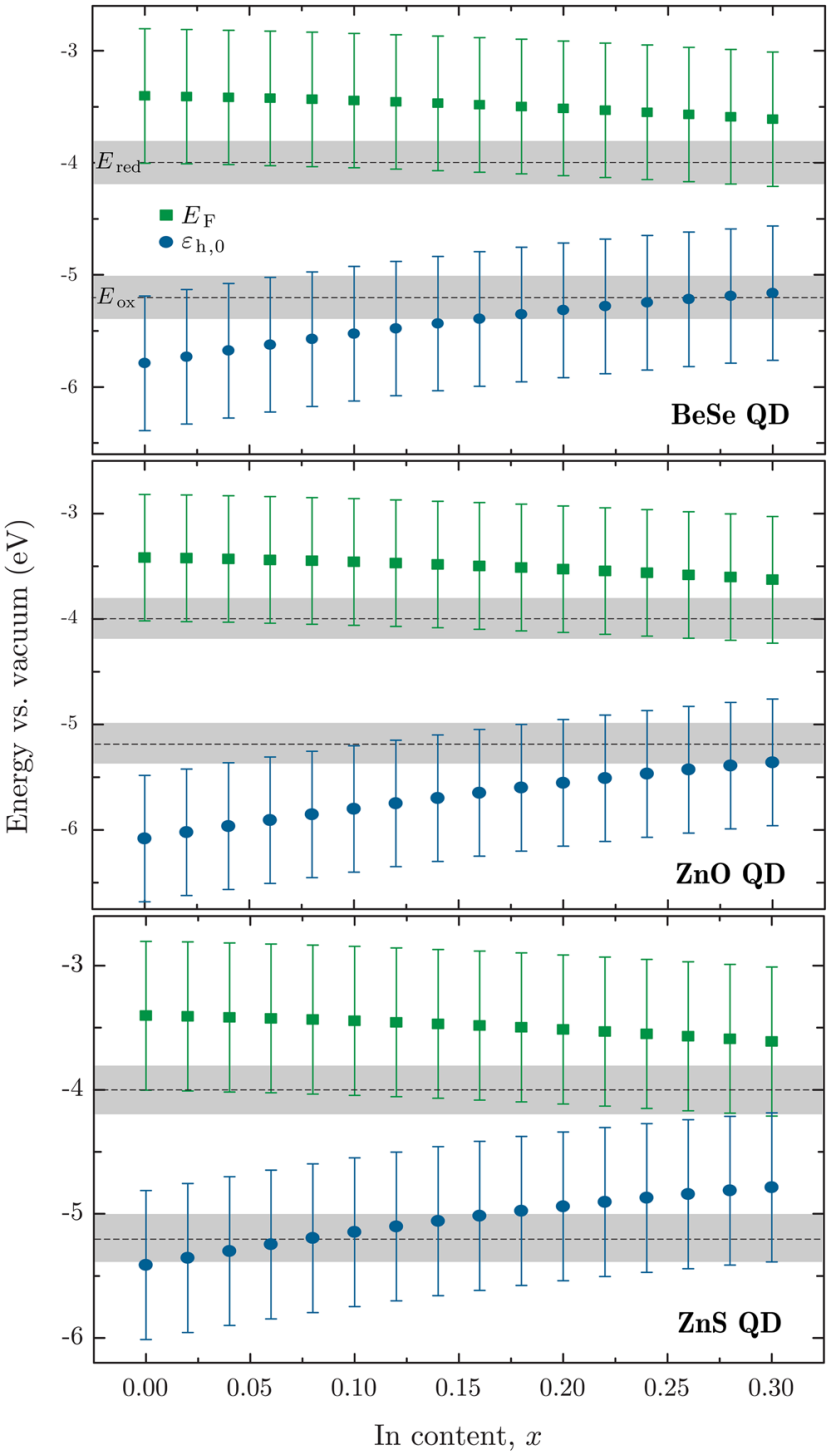
Fermi level *E*_F_ and first confined hole state *ε*_h,0_ for BeSe, ZnO and ZnS QDs on In_*x*_Ga_1−*x*_N with varying *x*. Hydrogen- and oxygen-production potentials, *E*_red_ and *E*_ox_, are shown as dashed lines, with their associated uncertainty represented by the shaded grey areas.

The absolute positions of *E*_F_ and *ε*_h,0_ relative to the vacuum, are shown in Fig. 7, alongside the hydrogen- and oxygen-production potentials *E*_red_ and *E*_ox_. Uncertainties, stemming from literature values for *χ* and the electrode–vacuum conversion potential, are shown as error bars or the grey band for *E*_red_ and *E*_ox_. As with Fig. 6, the absence of band bending means that energy levels are likely to be pushed slightly higher in energy in a real PEC.

The use of ZnO QDs is clearly the most promising: The confined hole state is consistently lower than for BeSe and ZnS, and ignoring uncertainties, stays below *E*_ox_ until over *x* = 0.3. Even with the oxygen-production requirement for an overpotential of 0.275 V, the confined hole state stays lower than *E*_ox_ until *x* = 0.18. For all three materials, *E*_F_ is higher than *E*_red_, though not within the uncertainties.

Previously, we speculated that the electron affinity of bulk GaN was higher than the average from literature values (~4 eV, rather than the average of 3.4 eV). If the higher value was used in Fig. 7, *E*_F_ would lie extremely close to *E*_red_. However, given only a small overpotential, of 0.050 V, is required for hydrogen production, the system could still plausibly split water. Furthermore, that bulk n-GaN has been shown to successfully split water in a PEC^24, 25^ highlights the likeliness that, at least for *x* = 0, *E*_ox_ is sufficiently higher in energy than *ε*_h,0_. Of course, a higher electron affinity would be beneficial for oxygen production, meaning the confined hole states would be at lower energies.

Including band bending at the SEI into the model would seem the next logical step, however we have already established that uncertainties in the Schottky barrier height are large, rendering results from its inclusion almost meaningless; we can create a variety of different systems that both can and cannot split water within the given uncertainties. Furthermore, the formation of band bending at a SEI with surface QDs is undocumented in literature, and the exact form that the Schottky barrier will take is not clear. Instead, we shall limit ourselves to a discussion of band bending at the SEI and present a few illustrative results for an “ideal” material system.

In regions of the semiconductor surface where there are no QDs, it seems reasonable to presume that the Schottky barrier formed will be equivalent to as if the electrode was a bulk semiconductor electrode. Small nanostructures are not large enough to form a space-charge region^29^, and as such, band bending is negligible. Surface QDs are likely to be small enough such that this is the case, and therefore the Schottky barrier height will certainly not be that of the QD material. The process of band bending is induced by the flow of carriers from the electrode to the electrolyte, and in the case of an n-type electrode, it is a flow of electrons that cause the bending^30, 31^. The presence of QDs creates a potential barrier for electrons, making it energetically favourable for them to transfer to the electrolyte via the surface of the bulk semiconductor, rather than by tunnelling through the barrier. Effectively, the QDs have no impact on the band bending and the interface between a QD and the electrolyte is *not* a typical SEI; that is, a Schottky barrier does not form as there is no flow of electrons from the dot to the electrolyte.

Modelling this interface at the surface of the QD could take two conceivable forms; either no contact at all is specified and a potential barrier is placed in the way, or a Schottky contact could be used whose height is simply the combination of the bulk Schottky barrier height and the QD potential barrier height as given from calculations without any contacts. The latter treats the QD as a perturbation to the bulk band bending and can be used to illustrate an “ideal” band structure. The key word here is *illustrate*, and in the following, parameter values are cherry-picked (within the previously discussed uncertainties) to produce the most optimal band structure.

**Figure 8:**
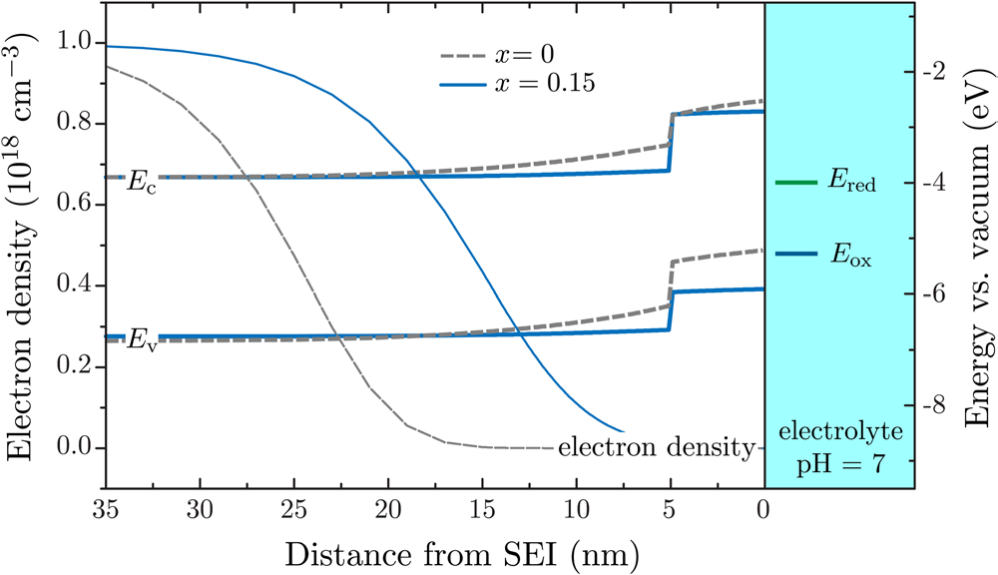
An illustration of the desired band structure and electron density, calculated by choosing the optimal parameter values within the uncertainties from literature values.

Starting with the desire for the Fermi level to be as close as possible to the hydrogen-production potential, while ensuring an overpotential of 0.050 V, gives us a value for the electron affinity of 3.9 eV. Choosing a higher In content *x* means a higher flat-band potential and thus a smaller Schottky barrier, while choosing a smaller *x* means less efficient absorption of the solar spectrum. For the case of *x* = 0, assuming the flat-band potential given by literature of −0.9 V, we obtain a Schottky barrier height of *ϕ*_B_ = 0.41 eV. On the other hand, if *x* = 0.15 (using a shift of ~0.14 V per 10% In content increase^10^ to give *V*_fb_ = −0.7 V), the Schottky barrier height is only *ϕ*_B_ = 0.2 eV. ZnO has proven itself to be the most desirable QD material and as such is chosen here. Figure 8 shows the resulting band structure, along with the electron density, for both of these cases. The band bending has resulted in the separation of carriers such that the electron density is negligible near the SEI, while the hole is confined in the QD. Coulomb interaction between electrons and holes is included within our model, and thus it is reassuring that even the small band bending illustrated in Fig. 8 is enough to split up the photogenerated carriers. The large Schottky barrier (*x* = 0) means a more effective splitting up of carriers, but potentially to the detriment of pushing the confined hole ground state too high, such that the requirement for a 0.275 V overpotential isn’t met.

The size of the QDs, as well as the substrate orientation, is likely to have an effect on confinement potential offered by the dots. We have chosen to exclude these parameters from our model as they will only serve to add further variability to results that already contain a considerable amount of uncertainty.

## Discussion

We have demonstrated that the novel use of nanostructures at the SEI to form a type-II heterojunction, in an attempt to increase the maximum photovoltage, can plausibly split water using ZnO QDs grown on n-InGaN with low In content. To the authors’ best knowledge, this system has never been investigated either theoretically or experimentally, and there is therefore a huge scope for further work to expand upon the results presented here.

The effectiveness of ZnO/InGaN in part depends on how much In can be incorporated before *E*_F_ is pushed to a lower energy than *E*_red_, or *ε*_h,0_ raises above *E*_ox_. The band gap of wurtzite GaN is 3.51 eV^28^, corresponding to 352 nm, a wavelength too small to absorb the majority of solar radiation. For In_0.15_Ga_0.85_N, this decreases to 2.92 eV (425 nm), for In_0.3_Ga_0.7_N it is 2.40 eV (517 nm) and for In_0.5_Ga_0.5_N it is as low as 1.80 eV (689 nm). The peak in solar radiation is a little over 500 nm, and thus it is highly desirable that an In content of approaching (or better, exceeding) *x* = 0.3 achieves water splitting. Our results demonstrate this may be possible, within uncertainties, for ZnO/InGaN, but experimental verification is needed to provide a definitive answer.

A common approach to increase the likelihood of electron- and hole-donating states straddling the hydrogen- and oxygen-production potentials is to use a tandem or multi-junction cell design^7^, and although the idea of utilising nanostructures at the SEI may ultimately negate the need for these more complex designs, there is no reason why such a nanostructured electrode couldn’t be used in future multi-junction PEC research.

Stability is, of course, another key factor. III-nitrides, including InGaN, have a good reputation for their photo-stability^10, 16, 17^. In general, p-type semiconductors are seen to be more stable than n-type, but as discussed earlier, the type-II system required would be an electron-confining one, which is much more difficult to achieve using InGaN as the bulk semiconductor. Furthermore, it should be noted that “good stability” in literature usually refers to the measuring of the photocurrent density over a period of hours, or at most, days^4, 32^, rather than the months or years which is of course desirable for a commercially-viable photoelectrolytic system. Therefore, the use of other techniques to improve stability (as discussed in, e.g., ref. 33) will be imperative.

The availability and Earth-abundance of the materials used to fabricate electrodes must also be taken into consideration, as well as any subsequent processing costs. Both Ga and In are scarce elements, at least in terms of availability, and thus the ideal photoelectrolytic system should avoid their use^34^. However, this should not deter us from their use in research toward a proof-of-concept; if the system we propose proves photoelectrolytically viable, then further research innovation (e.g., the development of new materials) will expand on our work to make it environmentally and economically viable. An example of this in action is the development of *copper zinc tin sulphide* as an Earth-abundant and thus cheaper alternative to CdTe and copper indium gallium selenide^34^.

The final caveat to the potential of ZnO/InGaN is whether or not such a system can be easily fabricated. It is encouraging that, though InGaN itself is regarded as a challenging material to grow^16, 35^, the fabrication of ZnO QDs on GaN has been demonstrated in literature by sintering^36^.

To conclude, we have proposed the use of type-II heterojunction formed by surface QDs for efficient water splitting under intense solar illumination, and have developed a theoretical model of a semiconductor electrode in a photoelectrochemical cell to assess the suitability of a wide range of substrate and QD material combinations. We find that ZnO QDs grown on n-InGaN is the most promising system, meeting the requirements that *E*_F_ − *ε*_h,0_ > 1.8 eV for *x* < 0.26, *E*_F_ > *E*_red_ for *x* < 0.3 and *ε*_h,0_ < *E*_ox_ for *x* < 0.3, excluding uncertainties and without band bending. However, large uncertainties plagued our attempt at the inclusion of a Schottky barrier at the SEI: within uncertainties, ZnO/InGaN QDs can be shown to be able to, and to not be able to, split water. Nonetheless, it is reassuring that a system capable of splitting water more than lies within said uncertainties. Ultimately, experimental work will prove whether this is the case or not, and could provide invaluable feedback for the further development of the model.

## Methods

Calculations were performed using the nextnano software package^37^, which solves the 8-band **k** · **p** Schrödinger, Poisson and drift–diffusion equations self-consistently, taking strain into account, giving us information about the energy levels and current distribution in a specified system. Fig. 3 shows an example of the 2D semiconductor electrodes modelled: The bulk substrate material is back-contacted with an ohmic contact (that in a real PEC would be connected to the counter electrode), and on the surface of the bulk material is a 10-nm-diameter, 5-nm-high QD, which is in turn contacted by a Schottky junction to mimic band bending at the SEI. The dimensions of the bulk semiconductor electrode material are likely to be much larger in practice (i.e., in the order of mm), however the 100 nm chosen is sufficiently large that the substrate’s size doesn’t affect the results.

The Schottky barrier requires, as an input, the Schottky barrier height *ϕ*_B_ = *E*_c,SEI_ − *E*_F_. Rearranging equation (3) and substituting it into *ϕ*_B_, we obtain:

4 $$\begin{array}{rcl}{\varphi }_{{\rm{B}}} & = & -e{V}_{{\rm{fb}}}-{E}_{{\rm{F}}}+{E}_{{\rm{c}}}-{E}_{{\rm{F}}}\\  & = & {-e{V}_{{\rm{fb}}}|}_{{E}_{{\rm{vac}}}=0}+\chi +2\alpha \end{array}$$

where $\chi ={-{E}_{c}|}_{{E}_{vac}=0}$ is the electron affinity, ${|}_{{E}_{vac}=0}$ indicates that a particular value is taken relative to the vacuum level (as opposed to, e.g., relative to a reference electrode), and *α* = *E*_c_ − *E*_F_ can be calculated by ref. 38

5 $$\begin{array}{c}\alpha ={k}_{{\rm{B}}}T\,\mathrm{ln}(\frac{{N}_{{\rm{c}}}}{n}).\,\end{array}$$

Here, *k*_B_ is the Boltzmann constant, *n* is the electron density and *N*_c_ is the effective density of states of the conduction band, which in turn can be calculated by

6 $$\begin{array}{c}{N}_{{\rm{c}}}=2{(\frac{2\pi {m}_{{\rm{e}}}^{\ast }{k}_{{\rm{B}}}T}{{h}^{2}})}^{\frac{3}{2}},\end{array}$$

where ${m}_{e}^{\ast }$ is the electron effective mass and *T* is the temperature^38^. We hence have a value for the Schottky barrier height that depends on the material parameters *V*_fb_, *χ*, ${m}_{e}^{\ast }$ and *n*, which for most semiconductor materials are easily found in literature, or in the case of *n*, depends on the doping during the growth of the samples and we can assume *n* ≈ *N*_*d*_ for sufficiently large doping densities^38^, where *N*_d_ is the dopant density. Unless explicitly stated, parameters are taken from refs 28, 38 and 39.

### Data availability

The datasets used in generating the figures are available at DOI https://dx.doi.org/10.17635/lancaster/researchdata/151.
